# A Review of Genetic Diversity Based on the Y Chromosome in the Romanian Population

**DOI:** 10.7759/cureus.67593

**Published:** 2024-08-23

**Authors:** Ramona Hodișan, Dana C Zaha, Claudia Jurca, Codruta D Petchesi, Marius Bembea

**Affiliations:** 1 Doctoral School of Biomedical Sciences, University of Oradea, Oradea, ROU; 2 Department of Preclinical Disciplines, University of Oradea, Faculty of Medicine and Pharmacy, Oradea, ROU

**Keywords:** haplogroup, haplotype, romanian population, human, y chromosome, genetic diversity

## Abstract

Y chromosome analysis is used in a number of practical applications, including investigations of criminal cases, establishment of paternity, searching for missing persons, studies on human migration, evolutionary research, and historical and genealogical investigations. Questions about the origin of individual ethnic groups are addressed not only through archaeological, linguistic, and ethnographic methods but also through molecular genetics methods. The study of genetic diversity in Romania is particularly interesting from several perspectives because Romania, located in Southeast Europe, is distinguished by the fact that the Carpathians and the Danube served as natural barriers against the migrations of peoples for centuries, thus influencing the genetic mixture of the population. This is relevant for understanding the history and formation of ethnic groups in the region. In addition, many ethnic minorities live in Romania, which adds an additional dimension of genetic and cultural diversity. This article aims to provide an updated picture of the genetic diversity in Romania and to highlight the significant studies carried out among the Romanian population. By analyzing the articles published in the Web of Science, Scopus, or PubMed databases, which explore genetic diversity using the Y chromosome, the aim is to better understand the current genetic panorama in Romania.

## Introduction and background

The human Y chromosome is distinguished by having the most extensive non-recombinant DNA sequence in the entire human genome. It is transmitted entirely from father to son, thus preserving a unique record of the history of paternal descent [1]. Usually, men carry a single Y chromosome, which is transmitted on the paternal line without changes in haplotypes, as long as they are located in the non-recombined region of the Y chromosome and have a low mutation rate [2].

Short tandem repeat (STR) genetic markers can be divided into short reps in autosomal tandem (A-STR), short tandem repetitions of the Y chromosome (Y-STR), and short tandem repeats of the X chromosome (X-STR), depending on their place on the chromosomes. This genetic diversity specific to sex chromosomes makes them particularly useful in identifying victims in cases of mass disasters, investigating family lineages in missing persons cases, and testing mixed evidence in sexual assault investigations [3].

STR loci are markers that help determine an individual's haplotype. Men who are closely related share similar haplotypes, except in cases of homoplasy [4]. Y-STR markers are haploid and do not undergo recombination except for pseudoautosomal regions [5].

Y-STR and single nucleotide polymorphisms (Y-SNP) present on the Y chromosome are high-value genetic markers used in population genetics studies and forensic investigations. These markers present distinctive inheritance patterns that facilitate the identification of paternal progeny and reconstruction of the male bloodline [6]. Y-STR loci are variations of DNA that consist of repeated sequences of 2-6 base pairs. These repetitions are often unique to each individual, which gives them a great capacity for discrimination when analyzing DNA from different population groups [7].

The traditional method of analyzing STR involves the use of capillary electrophoresis to distinguish between alleles based on the length of the fragment. These alleles can reach the same length through a variety of mutations, meaning that each length-based category reflects a diverse set of alleles with different evolutionary and mutational histories. With the use of parallel massive sequencing, this fact becomes more obvious, because this method allows the resolution of STR alleles of the same length (called isometric alleles) by detecting repeated pattern variation within networks and identifying single nucleotide polymorphisms (SNPs) and indels, in both flanking and repeated sequences [8].

In the last two decades, multiplex systems have been developed by adding other markers, starting with a minimum set of nine Y loci haplotypes [9]. This selection of markers was made in accordance with the general selection criteria proposed by international working groups, such as the International Society for Forensic Genetics (ISFG) and Scientific Working Group on DNA Analysis Methods (SWGDAM), with the hope of improving the global capacity for discrimination. Currently, there are several validated commercial kits that include 22-25 Y markers, including those that consist of both Y-STRs and Y-STRs with rapid mutations (RM Y-STRs), which can be genotyped using both capillary electrophoresis and massive parallel sequencing methods [9].

A general limitation of Y-STR genotyping is its reduced ability to exclude paternal relatives as potential contributors to DNA evidence. Since Y-STRs are located in the non-recombinant region of the Y chromosome, paternal relatives will share identical haplotypes in the absence of mutational events. Differentiation between men also becomes a challenge in cases of inbreeding events, population isolation, and patrilocal practices that reduce Y chromosome diversity within a population [10]. Improving discriminatory Y-STR capacities through capillary electrophoresis techniques is currently achieved through two main approaches: the use of markers with higher mutation rates and the development of higher Y-STR panels. However, capillary electrophoresis techniques based on allele size discrimination cannot detect the sequence variation that may exist between alleles of the same size (isometric) [10].

SNPs from non-recombining regions of the male-specific Y chromosome were used to develop a solid phylogenetic tree of its haplogroup, which is extensively used to distinguish between populations and for evolutionary studies, genetic structure analyses, and the deduction of bio-geographic origin [11].

Modern population studies are frequently based on genomic analyses, mainly using SNP microarray technology. This method can identify variants associated with diseases or traits and is essential for the advancement of personalized medicine or forensic investigations [12]. These methods allow samples to be genome-genotyped and new SNP markers to be searched for in the different haplogroups of the Y chromosome. The use of new specific and highly informative SNP markers of the Y chromosome is one of the most promising tools for analyzing gene groups in regional and ethnic populations [13]. Progress in massive parallel sequencing methods has led to the analysis of population gene pools at the maximum possible level of resolution. The discovery of a considerable number of new information markers has allowed the phylogenetic relationships of individual lines within haplogroups to be investigated with an accuracy that was not previously achievable. Genotyping new SNP markers can serve not only as an addition but also as an alternative to identifying DNA through autosomal loci. It is also possible to develop effective test systems to determine the most likely ancestral origin of an individual [13].

Researchers often resort to examinations of short tandem repeat patterns of the Y chromosome to understand the phylogenies of the paternally inherited portion of its non-recombining region. Because they exhibit a higher mutation rate, they provide more precise typing resolution than biallelic polymorphisms, which evolve more slowly [12].

Y chromosome testing has a variety of practical applications, including forensic investigations, paternity determination, analysis of male/female DNA mixtures, missing persons investigations, human migration studies, evolutionary research, and historical and genealogical investigations [14-16]. Questions about the origin of individual ethnic groups are addressed not only through archaeological, linguistic, and ethnographic methods but also through molecular genetics methods [17]. The main applications of Y-STRs have a similar applicability and the same properties are also relevant in molecular anthropology, where Y-STR variability can be used to estimate relationships between different populations and individuals, contributing to the interpretation of human history [18].

Currently, there are extensive and ever-expanding databases dedicated to estimating the frequencies of the Y-STR haplotype among various human populations or ethnic groups around the world [19]. The main important database for the Y chromosome is the Y-Chromosome STR Haplotype Reference Database (YHRD) (https://yhrd.org/). YHRD centralizes national databases to meet the requirements of a local Y-STR refining database according to legislative requirements. This is a reference database of Y chromosome haplotypes, available free of charge, that aims to decipher the differences between populations, specific to the Y chromosome, and quantify the effects on the frequency estimation process [20].

Romania is an independent state located in Southeast Europe, north of the Balkan Peninsula, bordering Ukraine to the north, the Republic of Moldova to the east, Bulgaria to the south, Serbia to the southwest, and Hungary to the west. It is in the lower basin of the Danube, which exits to the Black Sea to the southeast, and has its relief focused on the arch of the Carpathians, with the Transylvanian Plateau in the center and the Carpathians, to the east, south, and west. 

According to [21], Romania has a population of 19,053,815 inhabitants, of which 9,245,540 are male and 9,808,275 are female. Along with the main ethnic group, the Romanians, 20 other ethnic communities live on the territory of Romania. The most important ethnic minority community are Hungarians, who represent 5.26% of the total, followed by Roma 2.99% and Ukrainians 0.24%. Other ethnic groups are Germans, Turks, Russian-Lipovans, Tatars, Serbs, Slovaks, Bulgarians, Croats, Greeks, Italians, Jews, Czechs, Poles, Ruthenians, Armenians, Albanians, and Macedonians.

This study aims to enhance our understanding of genetic diversity by identifying and examining the literature focusing on the genetic diversity of the Romanian population through Y chromosome analysis. Its goal is to provide a synthesis of these research efforts and an updated evaluation of the literature, presenting the haplotypes and haplogroups prevalent in Romanian populations. 

## Review

Materials and methods

All studies carried out and communicated on population groups in Romania that focused on Y chromosome haplotype diversity were identified. Both studies based on the identification of Y-STR markers and those on the identification of Y-SNPs were eligible.

The specialized literature was selected from the following databases in December 2023: PubMed, Web of Science, Scopus, and YHRD. Articles with full text and relevant summaries that studied the haplotype diversity of the Y chromosome and were conducted in Romania, using study groups from the population of Romanian nationality, were selected. The search for relevant articles was performed using the keywords "Y Chromosome," "Romania," and "human" in PubMed, the Web of Science, and Scopus.

The criteria for inclusion in the study are as follows: (i) publications written only in English; (ii) publications that aim to study genetic diversity through human Y chromosome analysis; and (iii) studies that have analyzed populations in the territory of Romania. The exclusion criteria are as follows: (i) publications that have focused on the analysis of other species (animals, plants); (ii) studies that have been solely aimed at investigating mitochondrial DNA (mtDNA) and ancient DNA (aDNA); and (iii) publications that focused on areas such as clinical research.

Results and discussion

General Characterization of Publications

A total of 11 publications were analyzed, which had the main purpose of examining the genetic diversity of the population by analyzing the Y chromosome. Most of the articles were published between 2001 and 2006 (six articles), and the size of the study groups varied from 54 to 219 analyzed samples. The average number of study participants was approximately 130 men.

From the point of view of the areas, most studies were conducted in the center area of Romania (n=4), three studies were reported in the south of the country, two studies were from Eastern Romania, one study was in the northwest area, and one was a study with participants from all over the country, with the selection criterion being that they bore the name "Basarab." The research carried out so far in our country is presented in Table 1.

**Table 1:** Studies on genetic diversity based on Y chromosome analysis performed in Romanian populations

No.	Author(s)	Year of publication	Region	Study lot (no.)	Number of loci	Main results	Reference
1	Stefan et al.	2001	Romania and the Republic of Moldova	219	9 Y-SNP	It demonstrated that the geographic region of the Carpathians is a breakpoint in the genetic geography of Central Eastern Europe.	[22]
2	Barbarii et al.	2003	Bucharest, Romania	104	7 Y-STR	In total, 97 different haplotypes were observed, of which 92 were unique. Discriminative capacity was 93.26% and haplotype diversity was 98.87%.	[23]
3	Barbarii et al.	2004	Transylvania, Siebenburgen area	59	7 Y-STR	Typing results reveal an extensive variety of haplotypes among the Saxon population.	[24]
4	Beer et al.	2004	Transylvania, Corund	99	8 Y-STR	From 99 samples, 99 different haplotypes were found. The haplotype diversity was 0.9973±0.0021.	[25]
5	Bosch et al.	2006	South-East Romania Ploiești and Constanța	67	19 Y-STR	The haplotype diversity for the batches from Constanța and Ploiesti was 0.998±0.009 and 0.998±0.007, respectively.	[26]
6	Egyed et al.	2006	Transylvania, Miercurea Ciuc, and Lunca de Sus	175	12 Y-STR	From a total of 175 samples, 134 different haplotypes were observed. The haplotype diversity for the Szekler population was 0.9987±0.005, and for the Csango population, it was 0.9883±0.016.	[27]
7	Stanciu et al.	2010	South-East Romania	122	17 Y-STR	In total, 115 different haplotypes were determined and 109 were unique.	[28]
8	Bembea et al.	2011	North-West Romania	175	12 Y-STR	The haplotype diversity was 0.9931 in the Oradea batch, 0.2134 in the Tileagd batch, 0.9747 in the Şinteu batch, and 0.9536 in Palota.	[29]
9	Martinez-Cruz et al.	2012	Dolj, Mehedinți, Cluj, and Brașov	178	19 Y-STR	The group of individuals with the name "Basarab" has a haplotype diversity of 0.9286±0.027, and the haplotype diversities in the populations from Cluj, Brașov, Dolj, and Mehedinți are 0.9973±0.005, 0.9796±0.007, 0.9775±0.013, and 0.9818±0.046, respectively.	[30]
10	Varzari et al.	2013	East Romania	54	17 Y-STR	The haplotype diversity for the Romanian group was 0.9895.	[31]
11	Borbély et al.	2023	Transylvania, Odorheiu Secuiesc	92	23 Y-STR	The haplotype diversity for the Szekely population was 0.9995 and the proportion of single haplotypes was 97.8%.	[32]

Haplotype Diversity in Romanian Populations

In all the studies carried out in the center area, both ethnic communities and genetic isolates were analyzed. Two studies have presented genetic diversity of, respectively, the Szekely from Corund and the Szekely from Miercurea Ciuc and the Csangos from Lunca de Sus. The study [25] conducted in Corund, Romania, on a group of 99 unrelated men showed that the most common haplotype, using seven markers, is 14-23-13-31-12-10-11-13/13, with a frequency of 0.044, and its diversity is 0.9973±0.0021. A greater haplotype diversity, 0.9987±0.005, was observed in the Szeklers in Miercurea Ciuc [27]. In the study conducted on the populations of Miercurea Ciuc and Lunca de Sus, 134 different haplotypes were observed, and only six haplotypes were found in both populations (Szekely and Csango).

Studies conducted in the south and southeast of Romania have analyzed the diversity of the Y chromosome haplotype. One study [23] showed a haplotype diversity of 0.989 and a discriminatory capacity of 93.26%. An interesting fact which was found was that the most common haplotype in the European database was identified only once in a total of 104 samples.

The study carried out in nine counties located in the southeastern region of Romania analyzed a group of 122 unrelated men. By calculating the genetic distance and generating dendrograms, it showed that the Romanian male population is genetically similar to the populations of Macedonia, Serbia, Bosnia-Herzegovina, and Croatia [28]. Two populations from Ploiesti and Constanța were analyzed in the study by Bosch et al. in 2006 [26]. This research aimed to analyze the genetic diversity of two uniparental markers of the human genome, mtDNA and the Y chromosome, using their well-defined phylogenies to reveal population structure in the Balkans.

The first study conducted in 2001 [22] demonstrated that the geographical region of the Carpathians is a breakpoint in the genetic geography of Central Eastern Europe, providing a finer definition of one of the possible sudden genetic changes between Western and Eastern Europe. A representative study of the eastern region of Romania [31] showed that the Y STR haplotype diversity on 17 loci for the group from Piatra Neamț and Buhuși (n=54) was 0.9895. This study demonstrated that even if Romanians and Moldovans share cultural traits, the two peoples are not genetically similar.

The Most Common Haplogroups in the Romanian Population

**Figure 1:**
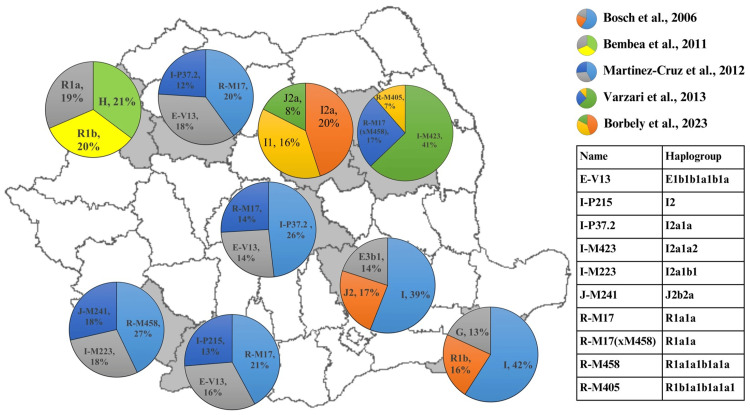
Distribution of the main three haplogroups in different regions of Romania The map of Romania was created in R, and the table was created according to the ISOGG Y-DNA Haplogroup Tree 2019-2020

Five different studies carried out in the territory of Romania investigated haplogroup diversity by analyzing the Y chromosome. These studies provide a detailed insight into the genetic composition of the Romanian population and the contribution of different paternal lines to the country's genetic heritage. Figure 1 shows the distribution of the main three haplogroups in different regions of Romania and an additional table for haplogroups created according to the ISOGG Y-DNA Haplogroup Tree 2019-2020 (https://isogg.org/tree/) [33].

The research carried out in North-West Romania [29] studied three genetic isolates from Bihor County. The study group was composed of 175 apparently healthy and non-related men, the Oradea control group (n=73), and three ethnic isolates: Şinteu (Slovak region, n=35), Tileagd (Roma population, n=35) and Palota (population with German origins, n=32). It was observed that the haplogroups determined in the Bihor population correspond to those in Romania. In the Şinteu isolate, the most common haplogroup is R1a, which is specific to Balto-Slavic populations. The Tileagd isolate was the most homogeneous: except for one individual, all others belonged to a single haplogroup, H, which is specific to the Indian subcontinent. In the Palota isolate, the dominant haplogroup, R1b, is characteristic of the German region, thus suggesting the origin of the inhabitants of this area.

In Transylvania, a study carried out with 23 Y-STR markers [32] analyzed 92 Y-STR and Y-SNP profiles of the Y chromosome. Hungarian-speaking people, born in the area of Odorheiu Secuiesc, with Szekler ancestors, and documented in that area for the second generation, were analyzed. Eight Y macro-haplogroups (E, G, H, I, J, Q, R, and T) were observed, with some subhaplogroups predominant in Inner Asian (R1a-Z93), South Asian/European Roma (H-M52), and Northern Eurasian (Q-M242) groups. 

Meanwhile, a study conducted in the Republic of Moldova compared three populations: Moldavians (from Sofia and Karahasani), Ukrainians (from Rashkov, Transnistria), and Romanians (from Piatra Neamț and Buhuși, Eastern Romania) [31]. It showed that the dominant haplogroup of the population from Piatra Neamț and Buhuși is I-M423, followed by R-M17*, R-M405, E-V13, and R-M412*.

The first genetic study on a surname of royal lineage carried out in Romania had as its study group 29 non-related men with the surname "Basarab" and 149 Romanians from different regions: Dolj, Mehedinți, Cluj, and Brașov [30]. A total of 131 SNPs from the non-recombined region of the Y chromosome were genotyped, and 19 loci were analyzed. All determined haplogroups are common in Romania and other Central Eastern European populations. The results of the study showed that not all those who bear the name "Basarab" are descendants of the dynasty of the first rulers of Wallachia with the same name.

Among the most common haplogroups in Romania are I2a, R1a, E1b1b, I1a, J2b, and H. Y haplogroup analysis shows that the most common haplogroup is I2a, as observed by seven study groups. Haplogroup I (I-M170) is a significant component of the European Y chromosome gene pool [34]. Subhaplogroups I1a and I2b are mostly found in Northern and Western Europe, and subhaplogroup I2a is most common in Eastern Europe and the Balkans [35]. I1a is predominant in Northern Europe, with the highest frequencies in Scandinavian populations; from its peak in Scandinavia, the frequency of subhaplogroup I1a gradually decreases towards both the Urals and the Atlantic periphery [34].

Another haplogroup often found in Romania is R1a, which was observed in six populations. The two most common descendant clades of R1 are R1a and R1b, and in Europe, haplogroup R1a is most common in the east, while the distribution of haplogroup R1b is more gradual, with an increase in frequency observed in Northwestern Europe [36]. This division is thought to reflect episodic population expansions during the postglacial period, including those associated with the establishment of agricultural and pastoral economies. More than 10% of men in a region stretching from South Asia to Scandinavia share a common ancestor within the R1a-M420 haplogroup, and most of them fall into the R1a1-M17/M198 subclades [37], while haplogroup R1a-M458 is found at frequencies approaching or even exceeding 30% in Eastern Europe [33].

Haplogroup R1a1a7 concentrates its spread in Central and Eastern Europe, not extending to the east beyond the Urals or to the south beyond Turkey. Central and southern Poland record the highest incidence of haplogroup R1a1a7, exceeding 30%. In contrast, the R1a1a*(xM458) chromosomes are less common in Poland but more common in Belarus and southwestern Russia. In Europe, the R1a1a7-M458 subclade dominates the variant of the haplogroup R1A1a, whereas it is absent in Asia. R1a1a7's European origin, narrow distribution, and demographic expansion suggest a successful spread during the early Holocene [38].

In the research carried out in Romania, haplogroup E1b1b (E-V13) was identified in three population groups, haplogroup J2b (J-M241) in another population, and haplogroup H in another distinct population. Haplogroup E1b1 is predominantly present in Africa, being the most widespread Y chromosome haplogroup in this region, and is notable for its internal diversity [39]. In contrast, haplogroup E1b-V13 is specific to the Balkan region, being the defining characteristic of that area [40]. The J2b (J-M241) haplogroup forms a network with a central haplotype and is most common in the southern Balkans, probably as a result of a rapid expansion that began in Neolithic times in the Anatolian region (Sea Minor) [41].

In the context of genetic diversity, genetically isolated populations are particularly interesting to study because they provide a unique perspective on human evolution in an isolated environment. Haplogroup H is characteristic of Roma populations but is commonly observed in populations in India, suggesting a possible migratory link from those regions to Europe in history [40,42].

## Conclusions

Determining the diversity of haplotypes by analyzing the Y chromosome can provide a wide range of information, including population history, migratory movements, and kinship links between individuals. Additionally, information can be obtained about past human migration routes and the geographic distribution of populations and their evolution by identifying haplogroups.

By analyzing the Y chromosome haplotypes, we can obtain a clearer picture of how the Romanian people and other ethnic groups in Romania are genetically connected to other populations in Europe and around the world. Currently, there are a small number of studies on genetic diversity based on the Y chromosome in Romania, with only 11 identified in the last two decades. These studies looked at a variable number of loci, ranging from 7 to 23, in different populations. Only five of these studies analyzed Y chromosome haplogroups. In Romania, I2a, R1a, and E1b1b stand out among the most frequent haplogroups, illustrating the complex diversity of the genetic heritage of the Romanian people.

This study of Y chromosome diversity in Romania's geographical and historical context provides a contribution to understanding the history and genetic identity of the Romanian people and the populations in the surrounding region. To provide a broader perspective on the genetic composition and genetic interactions between different ethnic groups, it is important to continue and expand research on genetic diversity in all regions of Romania, both through Y-STR analysis on a larger number of markers and through Y-SNP analysis.

## References

[1] Paz Sepúlveda PB, Mayordomo AC, Sala C (2022). Human Y chromosome sequences from Q haplogroup reveal a South American settlement pre-18,000 years ago and a profound genomic impact during the Younger Dryas. PLoS One.

[2] Liu Z, Long G, Lang Y, Liu D, Zhang B, Yu S, Guo F (2023). Sequence-based mutation patterns at 41 Y chromosomal STRs in 2 548 father-son pairs. Forensic Sci Res.

[3] Fu J, Song B, Qian J, He T, Chen H, Cheng J, Fu J (2023). Genetic polymorphism analysis of 24 Y-STRs in a Han Chinese population in Luzhou, Southwest China. Genes (Basel).

[4] Ashirbekov Y, Seidualy M, Abaildayev A (2023). Genetic polymorphism of Y-chromosome in Kazakh populations from Southern Kazakhstan. BMC Genomics.

[5] Neyra-Rivera CD, Ramos ED, Ingunza EG (2023). Analysis of 27 Y-chromosomal STR loci of the Mestizo Peruvian population. Egypt J Forensic Sci.

[6] Fu D, Adnan A, Yao J, Aldayan NH, Wang CC, Hongyi C (2023). Unraveling the paternal genetic structure and forensic traits of the Hui population in Liaoning Province, China using Y-chromosome analysis. BMC Genomics.

[7] Shabalala S, Ghai M, Okpeku M (2023). Analysis of Y-STR diversity and DNA methylation variation among Black and Indian males from KwaZulu-Natal, South Africa. Forensic Sci Int.

[8] Huszar TI, Bodmer WF, Hutnik K, Wetton JH, Jobling MA (2022). Sequencing of autosomal, mitochondrial and Y-chromosomal forensic markers in the People of the British Isles cohort detects population structure dominated by patrilineages. Forensic Sci Int Genet.

[9] Soldati G, Turrina S, De Leo D (2022). Forensic assessment on the application of a virtual pool of 30 Y-STRs. Forensic Sci Int Genet Suppl Ser.

[10] Kasu M, Fredericks J, Fraser M, Labuschagne C, Lesaoana M, D'Amato ME (2019). Novel Y-chromosome short tandem repeat sequence variation for loci DYS710, DYS518, DYS385, DYS644, DYS612, DYS626, DYS504, DYS481, DYS447 and DYS449. Int J Legal Med.

[11] Zhao GB, Miao L, Wang M (2023). Developmental validation of a high-resolution panel genotyping 639 Y-chromosome SNP and InDel markers and its evolutionary features in Chinese populations. BMC Genomics.

[12] Grochowalski Ł, Jarczak J, Urbanowicz M (2020). Y-chromosome genetic analysis of modern Polish population. Front Genet.

[13] Kharkov VN, Zarubin AA, Vagaitseva KV (2020). Y chromosome as a tool for DNA identification and determination of ethnoterritorial origin. Russ J Genet.

[14] Almohammed EK, Hadi A, Al-Asmakh M, Lazim H (2023). The Qatari population's genetic structure and gene flow as revealed by the Y chromosome. PLoS One.

[15] Mahdi Al-Zubaidi M, Arsheed Sabbah M, Khaleel Mahmood H (2023). Molecular diversity of 23-YSTR markers in Iraqi populations. Gene.

[16] Xu C, Wei W, Zuo M (2023). Genetic polymorphisms and phylogenetic characteristics of Tibeto-Burman-speaking Lahu population from Southwest China based on 41 Y-STR loci. Ann Hum Biol.

[17] Solovyev AV, Borisova TV, Romanov GP (2023). Genetic history of Russian old-settlers of the Arctic coast of Yakutia from the settlement of Russkoe Ust'ye inferred from Y chromosome data and genome-wide analysis. Russ J Genet.

[18] Boattini A, Sarno S, Mazzarisi AM (2019). Estimating Y-Str mutation rates and Tmrca through deep-rooting Italian pedigrees. Sci Rep.

[19] Nazir S, Adnan A, Rehman RA (2022). Mutation rate analysis of RM Y-STRs in deep-rooted multi-generational Punjabi pedigrees from Pakistan. Genes (Basel).

[20] Willuweit S, Roewer L (2007). Y chromosome haplotype reference database (YHRD): update. Forensic Sci Int Genet.

[21] (2023). RPL 2021. https://www.recensamantromania.ro.

[22] Stefan M, Stefanescu G, Gavrila L, Terrenato L, Jobling MA, Malaspina P, Novelletto A (2001). Y chromosome analysis reveals a sharp genetic boundary in the Carpathian region. Eur J Hum Genet.

[23] Barbarii LE, Rolf B, Dermengiu D (2003). Y-chromosomal STR haplotypes in a Romanian population sample. Int J Legal Med.

[24] Barbarii L, Constantinescu C, Rolf B (2004). A study on Y-STR haplotypes in the Saxon population from Transylvania (Siebenbürger Sachsen): is there an evidence for a German origin?. Rom J Leg Med.

[25] Beer Z, Csete K, Varga T (2004). Y-chromosome STR haplotype in Szekely population. Forensic Sci Int.

[26] Bosch E, Calafell F, González-Neira A (2006). Paternal and maternal lineages in the Balkans show a homogeneous landscape over linguistic barriers, except for the isolated Aromuns. Ann Hum Genet.

[27] Egyed B, Füredi S, Padar Z (2006). Population genetic study in two Transylvanian populations using forensically informative autosomal and Y-chromosomal STR markers. Forensic Sci Int.

[28] Stanciu F, Cuţăr V, Pîrlea S, Stoian V, Stoian IM, Sevastre O, Popescu OR (2010). Population data for Y-chromosome haplotypes defined by 17 STRs in South-East Romania. Leg Med (Tokyo).

[29] Bembea M, Patocs A, Kozma K, Jurca C, Skrypnyk C (2011). Y-chromosome STR haplotype diversity in three ethnically isolated population from North-Western Romania. Forensic Sci Int Genet.

[30] Martinez-Cruz B, Ioana M, Calafell F (2012). Y-chromosome analysis in individuals bearing the Basarab name of the first dynasty of Wallachian kings. PLoS One.

[31] Varzari A, Kharkov V, Nikitin AG (2013). Paleo-Balkan and Slavic contributions to the genetic pool of Moldavians: insights from the Y chromosome. PLoS One.

[32] Borbély N, Székely O, Szeifert B (2023). High coverage mitogenomes and Y-chromosomal typing reveal ancient lineages in the modern-day Székely population in Romania. Genes (Basel).

[33] (2024). Y-DNA haplogroup tree 2019-2020. https://isogg.org/tree/.

[34] Rootsi S, Magri C, Kivisild T (2004). Phylogeography of Y-chromosome haplogroup I reveals distinct domains of prehistoric gene flow in Europe. Am J Hum Genet.

[35] Sezgin E, Drosdak A, McIntosh C (2010). Examination of disease-based selection, demographic history and population structure in European Y-chromosome haplogroup I. J Hum Genet.

[36] Myres NM, Rootsi S, Lin AA (2011). A major Y-chromosome haplogroup R1b Holocene era founder effect in Central and Western Europe. Eur J Hum Genet.

[37] Underhill PA, Poznik GD, Rootsi S (2015). The phylogenetic and geographic structure of Y-chromosome haplogroup R1a. Eur J Hum Genet.

[38] Underhill PA, Myres NM, Rootsi S (2010). Separating the post-Glacial coancestry of European and Asian Y chromosomes within haplogroup R1a. Eur J Hum Genet.

[39] Trombetta B, Cruciani F, Sellitto D, Scozzari R (2011). A new topology of the human Y chromosome haplogroup E1b1 (E-P2) revealed through the use of newly characterized binary polymorphisms. PLoS One.

[40] Navarro-López B, Granizo-Rodríguez E, Palencia-Madrid L, Raffone C, Baeta M, de Pancorbo MM (2021). Phylogeographic review of Y chromosome haplogroups in Europe. Int J Legal Med.

[41] Karachanak S, Grugni V, Fornarino S (2013). Y-chromosome diversity in modern Bulgarians: new clues about their ancestry. PLoS One.

[42] Regueiro M, Rivera L, Chennakrishnaiah S, Popovic B, Andjus S, Milasin J, Herrera RJ (2012). Ancestral modal Y-STR haplotype shared among Romani and South Indian populations. Gene.

